# Global DNA methylation and chondrogenesis of rat limb buds in a three-dimensional organ culture system

**DOI:** 10.17305/bjbms.2021.6584

**Published:** 2022-02-21

**Authors:** Vedrana Mužić-Radović, Paula Bunoza, Tihana Marić, Marta Himelreich-Perić, Floriana Bulić-Jakuš, Marta Takahashi, Gordana Jurić-Lekić, Nino Sinčić, Davor Ježek, Ana Katušić-Bojanac

**Affiliations:** 1 Center of Excellence in Reproductive and Regenerative Medicine, School of Medicine, Zagreb, Croatia; 2 Hospital for Medical Rehabilitation of the Heart and Lung Diseases and Rheumatism-Thalassotherapia Opatija, Opatija, Croatia; 3 Department of Medical Biology, School of Medicine, Zagreb, Croatia; 4 Department of Communicology, Catholic University of Croatia, Croatia; 5 Department of Histology and Embryology, School of Medicine, Zagreb, Croatia

**Keywords:** Chondrogenesis, global DNA methylation, limb bud, development, rat, organ culture, *ex vivo*, *in vitro*, serum free, embryo

## Abstract

Although DNA methylation epigenetically regulates development, data on global DNA methylation during the development of limb buds (LBs) are scarce. We aimed to investigate the global DNA methylation developmental dynamics in rat LBs cultivated in a serum supplemented (SS) and in chemically defined serum- and protein-free (SF) three-dimensional organ culture. Fischer rat front- and hind-LBs at the 13^th^ and 14^th^ gestation days (GDs) were cultivated at the air-liquid interface in Eagle’s Minimal Essential Medium (MEM) or MEM with 50% rat serum for 14 days, as SF and SS conditions, respectively. The methylation of repetitive DNA sequences (short interspersed DNA repetitive element [SINE] rat ID elements) was assessed by pyrosequencing. Development was evaluated by light microscopy and extracellular matrix glycosaminoglycans staining by safranin O. On isolation, weak safranin O staining was present only in more developed GD14 front-LBs. Chondrogenesis proceeded well in all cultures toward day 14, except in the SF-cultivated GD13 hind-LBs, where safranin O staining was almost absent on day 3 that was associated with a higher percentage of DNA methylation than in SF-cultivated GD13 front-LBs on day 3. In SF-cultivated front-LBs, a significant methylation increase between the 3^rd^ and 14^th^ days was detected. In SS-cultivated GD13 front-LBs, methylation increased significantly on day 3 and then decreased. In older GD14 SS-cultivated LBs, there was no increase of DNA methylation, but they were significantly hypomethylated relative to the SS-cultivated GD13 at days 3 and 14. We confirmed that the global DNA methylation increase is associated with less developed limb organ primordia that strive toward differentiation in vitro, which is of importance for regenerative medicine strategies.

## INTRODUCTION

Limb development is the consequence of inductive interaction between ectoderm and mesoderm, with regulatory inputs that are incredibly complex. It must be regarded as a four-dimensional process where the signals from the three axes change with the progress of development [1]. Changes in expression in more than 2000 genes during mesenchymal stem cell chondrogenesis *in vitro* were discovered [2]. Regulation of gene expression necessary for developmental processes during embryogenesis, postnatal development, and even in tissues of adults depends on main epigenetic mechanisms such as DNA methylation [3], posttranslational histone modifications, and RNA interference [4]. DNA methylation occurs mainly in CpG dinucleotides and is catalyzed by DNA methyltransferases (DNMTs) that form 5-methylcytosine (5mC). At a gene promoter or enhancer sequences, DNA methylation is frequently associated with gene repression, correlating with the presence of inhibitory histone modifications that prevent the binding of transcription factors. Passive DNA demethylation may occur during cell replication, while active DNA demethylation is achieved by 10-11 translocation proteins (TET) and oxidative intermediates [5,6]. DNA methylation marks are mostly posed on cytosines of the promoter and enhancer regions but also on gene bodies or repetitive sequences. In most cases, DNA methylation within gene regulatory sequences stops gene transcription. DNA methylation of repetitive sequences ensures genome stability by suppressing the transposition of sequences to unpredictable positions within the genome that may cause serious consequences such as the malignant transformation of cells [6]. Embryonic stem cells that lack DNA methylation are blocked in the initiation of differentiation, and dynamic DNA methylation and demethylation occur during cell fate commitment and terminal differentiation [7]. Deregulation of DNA methylation may lead to developmental anomalies of mammalian limbs *in vivo* [8] or changes in developmental parameters of limb buds (LBs) grown *in vitro* [9]. In addition, it is implicated in the pathogenesis of osteoarthritis [10], while recently, DNA methylation profiling has been proposed for sarcoma classification [11].

Contemporary research on the development of various organs/tissues, done outside of living organisms (*ex vivo*), aims at the establishment of models for substitution of damaged tissues/organs in regenerative medicine or for screening of embryotoxic/anti-tumor substances [9,12-14]. *In vitro*, three-dimensional organ culture systems have an advantage over two-dimensional cell culture systems because of the presence of tissue interactions important for gene expression and presumably also for the regulatory activity of epigenetic mechanisms such as DNA methylation [11,12]. This is also true for LBs development that must be regarded as a four-dimensional process [1,9], where it is necessary to consider the tissue interactions and time points during development *in vitro*. In embryonic/fetal organ culture systems grown *ex vivo*, the impact of the maternal organism is avoided and the susceptibility to environmental factors such as the media composition may be directly assessed [15]. In such experimental systems, chemically defined synthetic culture media are usually supplemented with, for example, the serum or platelet lysates that are of a variable composition even between batches of the same origin. Omission of animal ingredients such as the fetal bovine serum is also highly recommended for growing cells/tissues for clinical application [16,17]. Chemically defined synthetic culture media should be more precise in research of the inherent developmental potential of cells/tissues grown *ex vivo*. Although such defined media culture conditions were described as “an unmet goal” [17], we did such research on experimental teratoma originating from the gastrulating embryo-proper [15]. In this system, biologically active substances exerted more clearly their activity in chemically defined serum- and protein-free media (SF) than in serum-supplemented media (SS) [18].

A recent review on osteogenesis and chondrogenesis states that the knowledge about DNA methylation dynamics during endochondral ossification and chondrogenesis is not as complete as the knowledge on its role in early development [19]. Our previous work on the rat as the mammalian representative , has shown that overall growth and cell proliferation at the 13^th^ gestation day (GD) LB grown *in vitro* with a DNA hypomethylating drug 5-azacytidine, were impaired but with no consequence on differentiation [9]. In our research, *in vivo* on the treatment of pregnant rat females during 12-13 GDs with 5-azacytidine, we assessed prolonged global DNA hypomethylation in LBs. Such a global DNA methylation decrease led to limb anomalies at GD20 associated with reactive oxygen species [8]. In a recently published research on chicken micromass *in vitro* culture, decreased global DNA methylation was correlated to the interdigital cell death [20].

Due to the results on the global DNA methylation during limb development being scarce, in this research, we aimed to associate for the first time the dynamics of global DNA methylation to GD13 and GD14 LB chondrogenesis in our organ culture three-dimensional system [9]. Moreover, we aimed to investigate such association in two types of culture media, that is, SS and chemically defined serum-free medium chemically defined serum-free medium (SF). Dynamics of global methylation was quantified by pyrosequencing rat identifier (ID) elements because they are widespread through the genome and sensitive to changes of microenvironment [21].

## MATERIALS AND METHODS

### Experimental animals

Three-month-old males and females of Fischer strain inbred albino rats were used. Male rats were put in a cage with females overnight, and the 1^st^ day of pregnancy was determined the next morning by the finding of sperm in the vaginal smear. Pregnant dames were euthanized with Xylapan (xylazine) 8 mg/kg weight and Narketan 100 mg/kg on the 13^th^ and 14^th^ days of pregnancy (GD13 and GD14), and LBs were microsurgically dissected from the embryos under the dissecting microscope.

### *In vitro* culture

LBs were plated on the lens paper put on the stainless steel grid of a disposable organ culture dish (Falcon 3037). Eagle’s Minimum Essential Medium (MEM) with Hank’s balanced salt solution was used alone for the chemically defined SF culture conditions or was supplemented with 50% rat serum (SF culture conditions). The cultures were kept for 3 or 14 days in an incubator with 5% CO2 and 95% air at 37°C.

### Histology

Cultivated LBs were fixed in St. Marie solution (96% alcohol and 1% glacial acetic acid) for 24 hours. They were dehydrated through an ascending series of alcohols (96% and 100% ethanol, each 2 × 20 minutes) and benzene (1 x 30 minutes). After paraffin embedding, paraffin blocks were serially sectioned (5 μm). After deparaffinization, routine hematoxylin-eosin (HE) staining was done or, after brief staining with hematoxylin, slides were rinsed with water, immersed in acidic alcohol (250 μl 36.5% HCl in 100 ml of 96% ethanol), incubated with 0.001% fast green (FCF) for 30 minutes, immersed in 1% acetic acid, and incubated with 0.1% safranin O dye. The sections were then rehydrated in an ascending series of alcohol solutions; 96% ethanol (twice for 2 minutes), 100% ethanol (2 minutes), and xylene (twice for 2 minutes), and covered.

For immunohistochemistry, Monoclonal Mouse – Anti-Proliferating Cell Nuclear Antigen (PCNA), Clone PC10 (M0879) with Labeled streptavidin-biotin kit, (K0609) Dakocytomation, LSAB^®^2 System-HRP, and P0397; streptavidin/HRP, all from DAKO, Glostrup, Denmark, were used. Polyclonal Rabbit Cleaved Caspase-3 (Asp 175) Antibody (# 9661; Cell Signaling Technology, Danvers, USA), with secondary biotinylated Anti-Rabbit IgG antibody – Biotin 028K4858 (B8895; Sigma Aldrich, Taufkirchen, Germany) was used as recommended by the manufacturer. A standard nonspecific antibody was used for negative controls (No. V 1617 mouse IgG1, DAKO).

### DNA isolation

At least six LB samples/group (total 144 samples) were deparaffinized by xylene (2 × 5 minutes) and then incubated in 100%, 95%, and 70% ethanol (3 minutes each) in water. DNA was isolated in TE buffer pH 9.0 with 0.1 mg/ml of Proteinase K and 0.25% of Nonidet P40 at 56°C for 24 hours. Samples were heated for 10 minutes at 95°C to inactivate proteinase K, spun, and the supernatant was frozen at −20°C. DNA quality and concentrations were assessed and measured with the NanoDrop ND-2000 spectrophotometer (NanoDrop Technologies, Wilmington, DE).

### Bisulfite conversion and polymerase chain reaction

One thousand nanograms of unpurified isolated genomic DNA were used for bisulfite conversion by EpiTect Plus DNA Bisulfite Kit (#59124; Qiagen). It includes a clean-up step so that purification of DNA was not necessary. PyroMark PCR Kit (#978703; Qiagen) was used for PCR amplification in the following conditions: 95°C for 2 minutes, 43°C for 90 seconds, and 72°C for 60 seconds for 40 cycles. Forward primer was 5’-GGGTTGGGGATTTAG-3’ and biotinylated reverse primer was 5’-AACCCAAAACCTTA-3’.

### Global methylation analysis by pyrosequencing

PyroMark Q24 Advanced System with PyroMark Q24 CpG Advanced Reagents (#970922; Qiagen) was used for the pyrosequencing reaction as recommended by Qiagen. 5’-GGGGATTTAGTTTAGTGGT-3’ was the sequencing primer for the rat ID element [21]. DNA methylation data were obtained and analyzed by the PyroMark Q24 Advanced Software.

Quality control of the data was determined during the pyrosequencing run. Quality control of bisulfite conversion for all samples was incorporated in the pyrosequencing assay. During assay design, the control dispensation of cytosine was generated at the non-CpG position. Since unmethylated cytosine should be converted to thymine, such control cytosine should not give a detectable signal [22]. The control of PCR reaction was conducted by running PCR blank, a reaction with all Master Mix components without the DNA. Samples obtained from PCR runs where blank control reactions did not show any product were considered for further pyrosequencing analysis. Moreover, the samples that passed quality control for bisulfite conversion after pyrosequencing were further analyzed.

### Ethical statement

The *in vitro* alternative to animal research *in vivo* was carried out with the permission of the Ethics Committee of the Faculty of Medicine, University of Zagreb (No. 04-76/2006-9), and according to the positive laws and regulations of the Republic of Croatia and EU directive 2010/63 on animal experimentation.

### Statistical analysis

In the analysis of the results, descriptive statistics was used to describe the measures of mean values and standard deviation. The normality of the data distribution was determined by the D’Agostino and Pearson Omnibus test. Due to non-parametric distribution, Kruskal–Wallis test with Dunn’s *post hoc* test was used for multiple comparisons in front- or hind- LBs during the cultivation time for each culture condition separately. Mann–Whitney U-test was used for comparisons between front- and hind-limb for each time point (days in culture) in the SF and SS cultivating conditions. Furthermore, the differences in the methylation status of GD13 and GD14 at a respective day of culture were also determined by Mann–Whitney U-test for SF and SS culture condition separately. Results with *p* values <0.05 were considered statistically significant.

## RESULTS

### Isolated LBs (GD13 and GD14)

**FIGURE 1:**
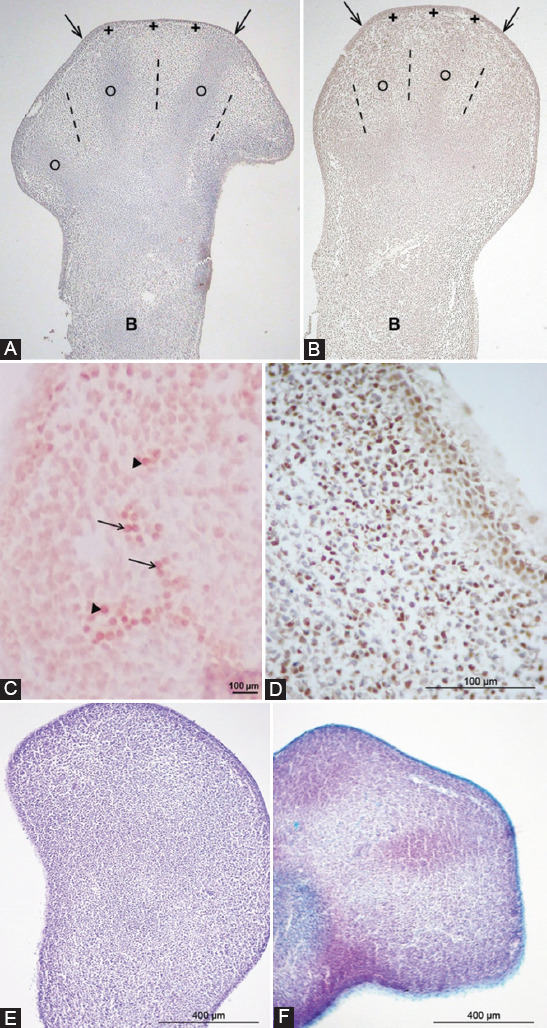
The anterior (A) and posterior (B) GD13 limb bud. x4. Immature epithelium (arrows); ray-like thickening of the mesenchyme (circle); future interdigital region (dashed line); future cartilage of wrist bones (asterisk); progression zone (plus); the base of the limb bud (B), HE. (C) Expression of cleaved caspase-3 in the mesenchymal cells of the GD14 anterior front limb bud; future interdigital region (arrows); future interphalangeal joint (arrowhead). Violet, ×200. (D) Expression of PCNA (brown) in mesenchymal cells of a GD14 hindlimb bud. Note negative internal control (blue). DAB, counterstained with hematoxylin. (E) No expression of GAGs in a GD14 hindlimb bud. Safranin O. (F) Expression of GAGs in a GD14 front limb bud. Safranin O. GD: Gestation day; PCNA: Proliferating cell nuclear antigen; GAGs: Glycosaminoglycans.

A typical form of the fins for front LB and paddle for hind LB was found in primary explants at both gestational stages (Figure 1A and B). The central part of the LB consisted of undifferentiated mesenchyme covered by a two-layer immature epithelium (ectoderm). In the peak part of the LB, there were ray-like thickenings of the mesenchyme, that is, the future bases of finger cartilage (slightly denser in 14-day-old embryos) surrounded by the loose mesenchyme in places of prospective interdigital regions. The described thickenings continued into thickenings of the future cartilage of the wrist bones (or feet in the hind-LB), surrounded by the loose mesenchyme at the base of the LB. In the hind-LB, the rays of the mesenchyme thickening were somewhat less common than in the anterior LB.

The expression of the cleaved caspase-3 (apoptotic marker) was assessed in only a few mesenchymal cells of the GD14 front-LB in the future interdigital region, and the interphalangeal joint (Figure 1C), and on their basis.

PCNA expression was detected in individual epithelial and mesenchymal cells (Figure 1D). Characteristic of the early stage of morphogenesis, the strongest PCNA signal was found in the horse-shoe progression zone (ZP) located subectodermally at the tip of the bud.

The explanted GD13 LBs and GD14 hind-LBs have not yet expressed glycosaminoglycans as the typical staining with safranin O had been negative (Figure 1E). In contrast, GD14 front-LBs were weakly stained by safranin O, indicating their more advanced development (Figure 1F).

### Chondrogenesis within rat LB in the 3D organ culture system

#### SS culture

LBs in the SS 2-week culture followed LB morphogenesis and tissue organization similar to those *in vivo*.

**FIGURE 2:**
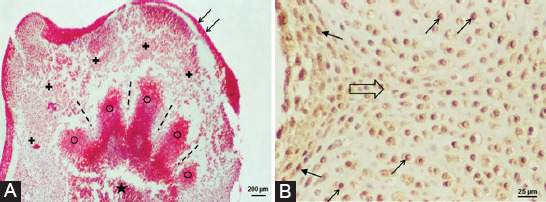
(A) Radial finger primordia (circle) in the anterior GD13 limb bud cultivated *in vitro* in the SS medium for 3 days. Interdigital region (dashed line), epithelium (arrow), central zone of decay (asterisk), mesenchyme (cross); Azan. (B) PCNA expression in the GD13 front limb bud cultivated for 14 days *in vitro*. PCNA signal in the mesenchymal cell (arrow); PCNA signal in the chondrocytes contained in the lacuna (thin arrow); direction of formation of the future interphalangeal joint (hollow arrow). GD: Gestation day; SS: Serum supplemented; PCNA: Proliferating cell nuclear antigen.

On day 3 of culture, the morphology of the LBs of ellipsoidal shape was retained, with no typical difference in the apical part for the anterior and posterior LBs. Direction of mesenchymal differentiation and condensation in conditions *in vivo* and *in vitro* began proximally at the base of the LB and continued distally toward the tip of the LB (Figure 2A). Larger cartilage bases were discovered at the base of the LB, which continued through the central decay zone into radial thickenings of the mesenchyme in the apex of the LB, that is, the bases of future fingers (Figure 2A).

After 14 days *in vitro*, the cartilage surrounded by the perichondrium was differentiated. Chondrocytes within the lacunas still expressed the proliferating cell marker, and the places of formation of future joint bases could be distinguished (Figure 2B). However, on the last day of culture, morphogenesis and differentiation within the LB did not reach the degree of differentiation *in vivo*. At that time, osteogenesis already occurs *in vivo* [8]. In other words, the gestation period in Fischer rats is 21 days, and the culture of GD13 and GD14 LB lasted for 14 days, which means that the last days of culture would fall in the neonatal stage.

**FIGURE 3:**
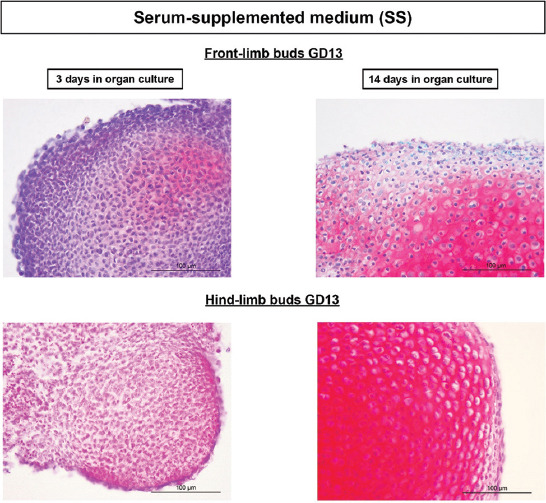
Progress of GD13 limb bud development/chondrogenesis in the SS medium. Note abundant, red-colored GAGs on the 3^rd^ day of culture in condensed mesenchyme, and intensely red, well-differentiated cartilage with lacunae after 14 days of cultivation. Safranin O. GD: Gestation day; SS: Serum supplemented; GAGs: Glycosaminoglycans.

**FIGURE 4:**
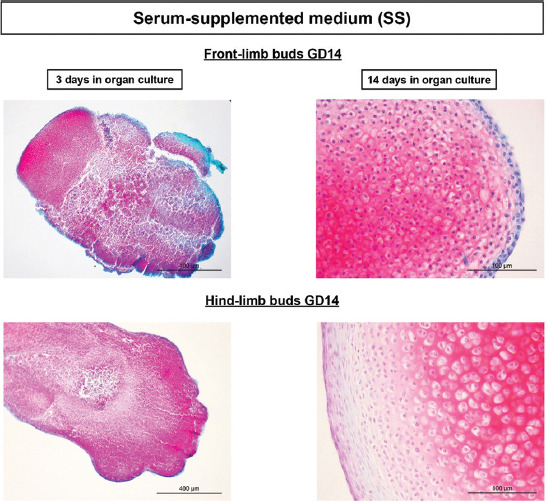
Progress of GD14 limb bud development/chondrogenesis in the SS medium. Note abundant, red-colored GAGs on the 3^rd^ day of culture in condensed mesenchyme, and intensely red well-differentiated cartilage with lacunae after 14 days of cultivation. Safranin O. GD: Gestation day; SS: Serum supplemented; GAGs: Glycosaminoglycans.

During the development of both GD13 and GD14 LBs in SS cultures, abundant safranin O staining has already appeared at day 3 of culture with the condensation of mesenchyme. The highest expression of glycosaminoglycans (safranin O most prominent red staining) was present at the end of the culture of both front- and hind-limb primordia that revealed well-differentiated cartilage with lacunae (Figures 3 and 4).

#### Serum-free culture

**FIGURE 5:**
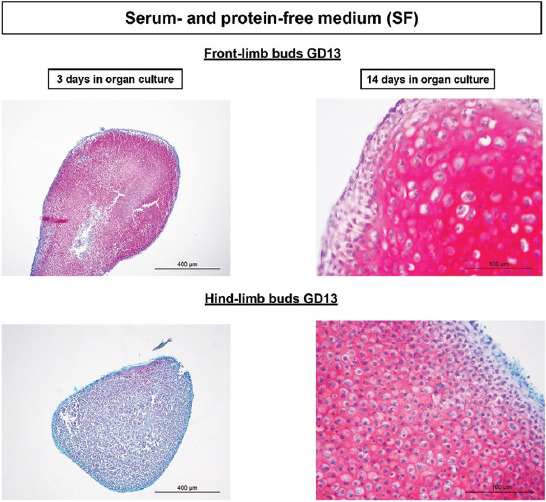
Progress of GD13 limb bud development/chondrogenesis in the chemically defined SF medium. Note that after 3 days in culture of hindlimb buds, red-colored GAGs are almost nonexistent in comparison to 3 days cultivated front limb buds. After 14 days of culture, such difference is not seen, and GAGs are abundantly expressed also in the cartilage with lacunae of hindlimb cultures. Safranin O. GD: Gestation day; SF: Serum and protein free; GAGs: Glycosaminoglycans.

**FIGURE 6:**
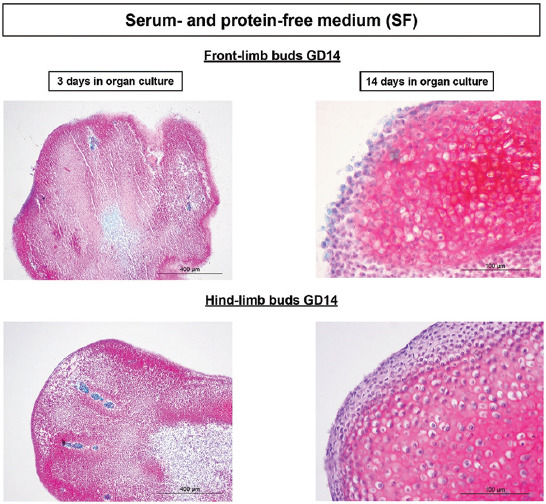
Progress of GD14 limb bud development/chondrogenesis in the chemically defined SF medium. Note abundant, red-colored GAGs on the 3^rd^ day of culture in condensed mesenchyme, and intensely red well-differentiated cartilage with lacunae after 14 days of cultivation. Safranin O. GD: Gestation day; SF: Serum and protein free; GAGs: Glycosaminoglycans.

In experiments with a metabolically poorer SF medium (Figures 5 and 6), the hindlimb GD13 stage development chondrogenesis was delayed. Namely, safranin O was almost not expressed on the 3^rd^ day of culture (Figure 5) in comparison to all other groups of LBs where it was abundant in areas with mesenchyme condensation (Figures 3, 4 and 6). However, differentiation of GD13 hind-LB progressed even in SF conditions, and glycosaminoglycans were abundantly expressed in the well-differentiated cartilage with lacunae on the last day of culture (Figure 5).

### Dynamics of global DNA methylation in LBs

#### Global DNA methylation in native and cultivated LBs of GD13 embryos

**FIGURE 7:**
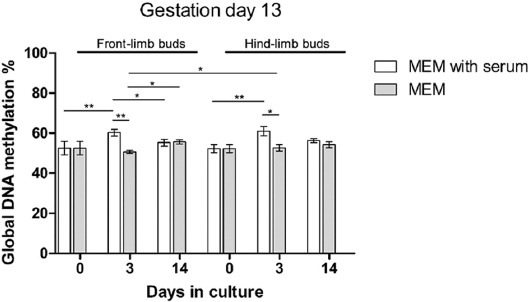
Dynamics of global DNA methylation in GD13 limb buds during *in vitro* cultivation. N=6 samples per group. Kruskal–Wallis test with Dunn’s *post hoc* test was used for multiple comparisons. Mann–Whitney U-test was used for comparisons of two values. *p* < 0.05, ** *p* < 0.001. GD: Gestation day; MEM: Minimal essential medium.

As we have shown a difference in safranin O staining between isolated and between some cultivated LBs, we were interested whether specific changes in global DNA methylation were associated to those findings. All results on the DNA methylation dynamics in GD13 are presented in Figure 7.

In native, isolated GD13 hind- and front-LBs, no safranin O staining was found, and the percentage of global DNA methylation was equal. In SS cultures, the percentage of methylation significantly increased to day 3 of culture in both front- and hind-LBs (*p* < 0.05) where safranin O staining had been detected in condensed mesenchyme. Therefore, global methylation increase was associated with the beginning of chondrogenesis. On day 14, where overt chondrogenesis took place, global DNA methylation decreased in SS cultures, significantly only for front-LBs (*p* < 0.05).

In SF conditions (Figure 7), a significant increase in methylation (*p* < 0.05) was found between days 3 and 14, only in the front-, but not in hind-LBs that were less developed at isolation. Moreover, in SF conditions, a significant difference (*p* < 0.05) in the DNA methylation was shown on the 3^rd^ day of cultivation between front- and hind-LBs. Namely, the average values of DNA methylation were 50.6% in front- and 52.67% in hind-LBs that developmentally lagged behind front-LBs at explantation. Importantly, higher DNA methylation in day 3 hind-LBs in SF culture was associated with a lower degree of chondrogenesis on day 3, as previously shown in Figure 5.

In both front- and hind-LBs, the degree of methylation was significantly lower on the 3^rd^ day of culture in SF than in SS-cultivated LBs that were associated with differences in media composition.

Global methylation of younger GD13 LBs cultivated *in vitro* exerted notable dynamic changes associated to the type of LB (front or hind), day of cultivation, and the culture media composition.

#### Global DNA methylation in native and cultivated LBs of GD14 embryos

**FIGURE 8:**
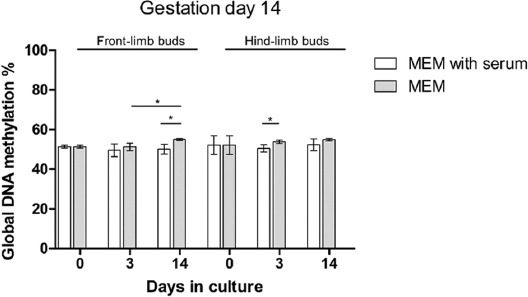
Dynamics of global DNA methylation in GD14 limb buds during *in vitro* cultivation. N=6 samples per group. Kruskal–Wallis test with Dunn’s *post hoc* test was used for multiple comparisons. Mann–Whitney U-test was used for comparisons of two values. * *p* < 0.05. GD: Gestation day; MEM: Minimal essential medium.

We were interested in whether older GD14 LBs will express similar global DNA dynamics as GD13. Results of global DNA methylation in GD14 LBs are shown in Figure 8. GD14 front- and hind-LBs showed no significant difference in global DNA methylation at the beginning or end of cultivation or between both culture conditions. However, in GD14 LBs cultivated in SF culture medium compared to those grown in SS culture medium, front- and hind-LBs showed higher methylation at day 14 and day 3, respectively. We also found a rise in global DNA methylation of SF-cultivated front-LBs from day 3 to day 14 of the culture.

We may conclude that the dynamics of DNA methylation in older GD14 LBs was not so prominent as in the younger GD13. In GD14, the differences in global methylation depended mainly on the type of the medium in contrast to GD13.

#### Comparison of global DNA methylation in cultivated LBs of GD13 and GD14 embryos

**FIGURE 9:**
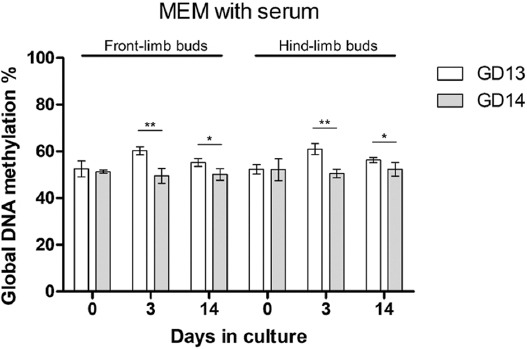
Comparison of global DNA methylation between two stages of limb buds (GD13 and GD14) cultivated in the SS medium. N=6 samples per group. Mann–Whitney U-test was used for comparisons of two values. * *p* < 0.05, ** *p* < 0.001. GD: Gestation day; SS: Serum supplemented; MEM: Minimal essential medium.

Finally, we compared global DNA methylation between two stages (GD13 and GD14) of isolated limb buds (Figure 9). The percentage of global DNA methylation was significantly higher in younger GD13 than older GD14 LBs. This was true for both front- and hind-LBs on days 3 and 14 of cultivation in SS culture medium, while in SF culture medium, such difference was not found (not shown).

## DISCUSSION

Our newly established LB *in vitro* experimental system shows that it is possible to achieve the chemically defined culture conditions, highly recommended for various *ex vivo* studies without any serum and other confounding supplements [17]. It enabled us to investigate the inherent developmental potential of LBs for chondrogenesis and its association with global DNA methylation. All *in vitro* cultivated explants of LBs differentiated well at the end of the 2-week culture period regardless of the stage of primary explantation or culture media used. For the first time, we showed the dynamics of global DNA methylation changes in an *in vitro* 3D organ culture model of mammalian LB development by comparing two successive developmental stages and two different culture media, that is, SS and chemically defined serum and protein free (SF). Our results show that the dynamics of the global methylation was associated with the age of explanted LBs, the progress of culture, and the type of medium.

We used a rat ID sequence to investigate global DNA methylation. The rodent ID family of repeats – SINE shows an evolutionary extremely high rate of amplification with 130,000 copies per haploid genome which are methylated to suppress their mobility and ensure genome stability. Therefore, averaging methylation across those repetitive sequences in the genome was recommended to quantify global methylation with high accuracy in basic epigenetic research [21], and we have successfully applied it before [8]. Pyrosequencing is an appropriate method for screening a large number of samples in the CpG-rich regions, compared to methods based on methylation-specific PCR [22,23]. Furthermore, among other contemporary DNA methylation assays, including immunostaining-based measurement of 5-methylcytosine, pyrosequencing showed the best all-around performance. Matched fresh-frozen and formalin-fixed paraffin-embedded samples showed highly similar results in methylation assays, and among absolute assays, pyrosequencing is optimal for highly fragmented DNA present in traces [24].

Safranin O staining used in this study represents an optimal method for the evaluation of chondrogenesis, for example, in cartilage grafts that repair osteochondral defects in large animals [25] or research on osteoarthritis [26]. Classical histological analysis of differentiation in samples and lack of safranin O staining of glycosaminoglycans in the primary explants of GD13 LBs confirmed that they developmentally lagged behind the GD14 LBs [27]. Therefore, light histology and safranin O staining complemented each other well in assessing the developmental progress of limb primordia. Transcriptomic analysis has shown differential expression of several thousands of specific genes during early mouse LB morphogenesis. The hindlimb identity may be achieved by inhibiting forelimb-specific genes [28], at least partly by DNA methylation. However, when we compared global DNA methylation in primarily isolated rat LBs of the same type (hind- or front-LBs) but of different developmental stages (GD13 and GD14), there was no statistically significant difference in the degree of global methylation. Therefore, the degree of development at early stages seems to depend more on the suppression of gene sequences by DNA methylation [28]. Indeed, DNA methylome appears to be established before the differentiation process, ensuring cell identity and genomic stability, and genomic regions undergo gains and losses of DNA methylation associated with activation/repression of lineage-specific genes and loss of pluripotency [7].

On the other hand, changes in global DNA methylation are also crucial for normal development [6,29-31]. The increase of global DNA methylation is associated with somatic morphogenesis and differentiation in mammals and in evolutionary distant plants [31]. Our results on developmental dynamics of the global DNA methylation have shown that such association is true for the continuation of development in younger, less developed LB (GD13), where the histological assessment showed no difference in optimal cartilage development after 14 days *in vitro*. As for the 3^rd^ day *in vitro*, only the younger hind-LBs in the SF medium lacked expression of glycosaminoglycans. This simple medium seems inadequate to provide conditions for phenotypic expression of the full LB developmental potential at this stage of development. Exactly in hind-LBs cultivated in SF medium, global DNA methylation was higher than in front-LBs cultivated in SF medium, which might be interpreted as if they were at the early stage of morphogenesis where DNA methylation is the prerequisite to proceed with the process of chondrogenesis.

In the optimal metabolic condition achieved in SS medium of GD13 LBs, the maximum global methylation was present on day 3 of culture in both anterior and posterior LBs and then declined. There was also a significantly higher level of global methylation in GD13 than in GD14 cultivated LB. This all agrees with the theory that global levels of DNA methylation increase during differentiation, although the expression of genes specific for a cell line, for example, chondrocytes, is associated with hypomethylation of genes typical of cartilage, mostly in enhancer regions [5].

Our whole organ, *ex vivo* cartilage differentiating system, represents a unique approach where we were able to associate the phenotypes during chondrogenesis with changes of the global DNA methylation. Most research on the epigenome in relation to the phenotype is still only devoted to associations because epigenomic signatures are complex and change with time in a cell-/tissue-specific manner [32]. Therefore, our *ex vivo* system might provide an optimal basis for further investigation of the gene-specific methylation during limb development and other epigenetic mechanisms that may influence DNA methylation [33,34].

## CONCLUSION

We may conclude that in our original 2-week organ culture system, early rat limb primordia develop further on, and well-differentiated cartilage is present at the end of the 2-week culture regardless of the explantation stage (GD13 or GD14) or the type of cultivation medium (SS or SF). However, during *in vitro* morphogenesis and differentiation, a specific increase of global DNA methylation level was more associated with less developed limb organ primordia (GD13) that strive toward differentiation, while the decline in global DNA methylation level was associated with more developed organ primordia (GD14). These results about the degree of global DNA methylation and differentiation may be important for novel strategies in regenerative medicine dealing with manufacturing cartilage grafts for osteochondral defects and a better understanding of diseases such as osteoarthritis, cancer, or developmental anomalies [8,25,35-37].
